# Geographic heterogeneity in the association between educational attainment and QUS-defined bone status among adults in Southwest China: a cross-sectional study

**DOI:** 10.1186/s12889-026-29217-9

**Published:** 2026-08-27

**Authors:** Xiao Zhang, Changchao Dong, Fen Yang, Yihang Xu, Sifan Hao, Jian Wang, Xing Zhao, Haojiang Zuo, Xiaofang Pei

**Affiliations:** 1 West China School of Public Health and West China Fourth Hospital, Sichuan University, Chengdu, Sichuan 610041 China; 2 Health Promotion and Food Nutrition & Safety Key Laboratory of Sichuan Province, Sichuan University, Chengdu, Sichuan 610041 China; 3 Innovation Center of Nursing Research, Nursing Key Laboratory of Sichuan Province, Sichuan University, Chengdu, Sichuan 610041 China

**Keywords:** Bone status, Educational attainment, Quantitative ultrasound, Regional heterogeneity, Southwest China, Path analysis

## Abstract

**Background:**

Although education is commonly regarded as a favorable factor for bone health, its association with bone status may vary across different socioeconomic and ecological contexts. We examined the association between educational attainment and QUS (quantitative ultrasound)-defined bone status across five provinces in Southwest China and explored province-specific indirect statistical associations.

**Methods:**

This cross-sectional study included 73,600 adults aged ≥ 30 years from Sichuan, Chongqing, Guizhou, Yunnan, and Tibet, recruited through a multistage, stratified cluster sampling design within the China Multi-Ethnic Cohort. QUS-defined bone status was classified as normal, reduced, and markedly reduced using calcaneal quantitative ultrasound-derived T-scores. Geographic effect modification was evaluated using cumulative logit ordinal regression. Province-specific path analysis was further conducted to explore potential indirect associations involving health behaviors, nutritional factors, and socioeconomic status. Partial proportional-odds models were applied in sensitivity analyses.

**Results:**

Participants had a mean age of 51.54 years (SD, 11.43), and 62.1% were women. Overall, 60.6%, 35.0%, and 4.4% of participants were classified into the normal, reduced, and markedly reduced QUS-defined bone-status categories. Education showed marked regional heterogeneity in its association with bone status (*P* < 0.001): higher educational attainment was associated with poorer bone status in Sichuan (β=-0.088, *P* < 0.05), Guizhou (β=-0.047, *P* < 0.05), and Tibet (β=-0.067, *P* < 0.05), better bone status in Chongqing (β = 0.045, *P* < 0.05), and no association in Yunnan (β = 0.015, *P* = 0.155). Path analyses showed distinct provincial patterns: higher alcohol consumption and higher dairy intake in Sichuan; higher income, higher dairy intake, and reduced smoking in Chongqing; lower physical activity in Guizhou and Yunnan; and no statistically significant indirect association was identified in Tibet. In PPOM sensitivity analyses, high (vs. low) educational attainment was associated with lower odds of worse bone status in Chongqing at both cumulative thresholds (OR = 0.738, 95% CI, 0.652–0.837; OR = 0.688, 95% CI, 0.541–0.875), higher odds of worse bone status in Guizhou (OR = 1.174, 95% CI, 1.018–1.352), and no clear association in Yunnan (OR = 0.873, 95% CI, 0.696–1.094).

**Conclusions:**

Educational attainment showed province-specific associations with QUS-defined bone status in Southwest China. Indirect statistical associations involving socioeconomic, behavioral, and dietary factors require confirmation in longitudinal studies.

**Supplementary Information:**

The online version contains supplementary material available at https://doi.org/10.1186/s12889-026-29217-9.

## Background

 As global population aging accelerates, osteoporosis has become a major public health concern worldwide [1]. Osteoporosis is characterized by low bone mass and microarchitectural deterioration, leading to increased bone fragility and a higher risk of fracture, particularly among postmenopausal women and older adults [2, 3]. Its clinical and public health significance lies largely in fracture-related disability, mortality, and healthcare burden, especially for hip fractures [4, 5].

China faces a particularly substantial burden of osteoporosis because of its large and rapidly aging population [6, 7]. According to the 2018 National Osteoporosis Epidemiological Survey, osteoporosis affected 19.2% of Chinese adults aged 50 years and older. Given the marked geographic, socioeconomic, dietary, and environmental diversity across China [8, 9], developing region-specific prevention strategies remains an important public health challenge.

In addition to biological factors, socioeconomic status has increasingly been recognized as an important determinant of bone health [10, 11]. Educational attainment, as a key social determinant of health, is often associated with better health awareness, healthier lifestyles, and improved access to health-related resources [12–15]. However, its association with bone status may not be uniform across populations and may be shaped by behavioral and contextual factors [16–20]. In China, the proportion of highly educated individuals has risen steadily in recent years [21], while technological and occupational changes have made sedentary work increasingly common among highly educated groups [22, 23]. These shifts suggest that educational advantage may not always translate into better bone health.

Most previous studies have examined the association between educational attainment and bone status within single regions, which may obscure important contextual variation [24, 25]. Southwest China, including Sichuan, Chongqing, Guizhou, Yunnan, and Tibet, provides a useful setting for examining such heterogeneity because these provinces differ markedly in socioeconomic conditions, ecological environments, sociocultural contexts, and access to health resources [26]. Based on prior evidence, we developed a hypothesized conceptual framework to organize the relationships among educational attainment, candidate intermediate variables, sociodemographic adjustment variables, geographic context, and bone status. Educational attainment was specified as the primary exposure and QUS-defined bone status as the outcome. Candidate intermediate variables were grouped into three domains: socioeconomic resources, represented by annual family income [10, 11]; health behaviors, including smoking, alcohol consumption, physical activity, and sleep disorder [20, 27]; and nutritional/metabolic factors, including calcium and vitamin D supplementation, dairy intake, and BMI [11, 17, 28]. Age, sex, ethnicity, and marital status were considered prespecified sociodemographic adjustment variables, whereas province was conceptualized as the primary geographic effect modifier.

Guided by this framework, we hypothesized that the association between educational attainment and QUS-defined bone status would vary across provinces. We therefore examined the overall and province-specific associations and used province-specific path analyses to explore this potential indirect association through the prespecified domains of candidate intermediate variables. Because all variables were measured at baseline, the resulting indirect estimates were interpreted solely as statistical associations and not as evidence of temporal ordering or causal mediation. The findings may provide a basis for future longitudinal research and for considering regional context when evaluating socioeconomic inequalities in bone health.

## Methods

### Study population and sampling framework

The present study used baseline data from the China Multi-Ethnic Cohort (CMEC), a community-based prospective cohort established in Southwest China. Participants were recruited using a multistage, stratified cluster sampling design [29]. Five provincial regions (Sichuan, Chongqing, Guizhou, Yunnan, and Tibet) were included to capture substantial geographic, environmental, and ethnic diversity. At the first stage, one to two settlements were selected for each targeted ethnic group, with particular consideration given to geographic, environmental, and socioeconomic diversity, including high-plateau, basin, rural, and highly air-polluted settings. At the second stage, one to eight communities within each settlement were selected by the local Centers for Disease Control and Prevention according to community size, migration patterns, local health conditions, and ethnic composition. At the final stage, eligible permanent residents in the selected communities were invited to participate through standardized community-based recruitment procedures, with consideration given to the local age and sex distribution [29]. The sampling frame at the individual level therefore comprised eligible permanent residents of the selected communities who were reached through community-based recruitment. No separate household-level listing or sampling procedure was conducted.

### Participants

**Fig. 1:**
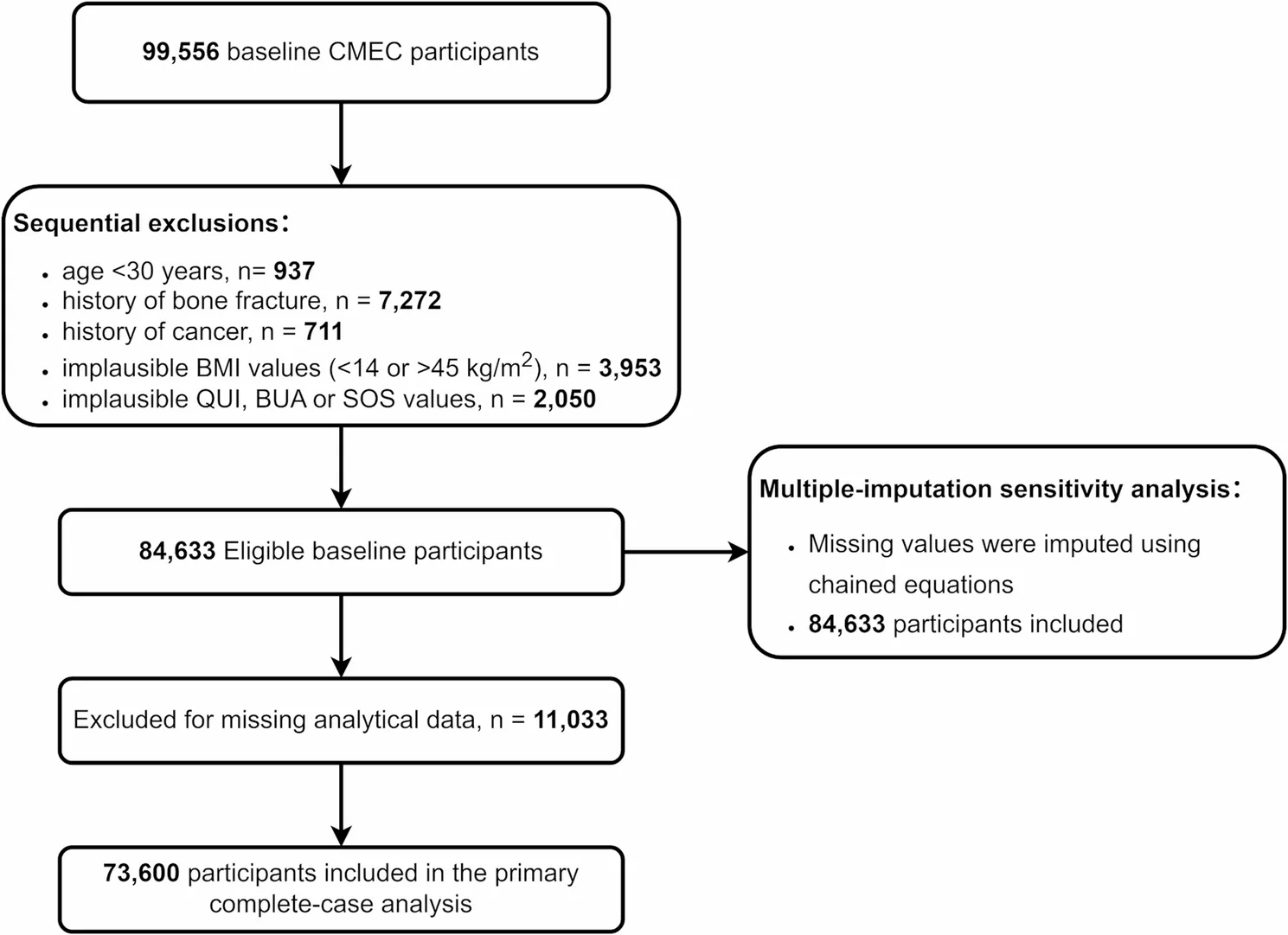
Flowchart of study participant selection

Eligible participants were permanent residents (≥ 6 months) aged 30 years or older from Sichuan, Chongqing, Guizhou, Yunnan, and Tibet, recruited between May 2018 and September 2019. Participants were included if they completed both the questionnaire and physical examination. Individuals with psychiatric disorders or impaired communication or comprehension were excluded. Sequential exclusion criteria included age < 30years, history of cancer or bone fracture, abnormal BMI (< 14 kg/m^2^ or > 45 kg/m^2^), abnormal calcaneus QUI values ( < − 35.95 or > 150.6), broadband ultrasound attenuation (BUA; <5 or > 60 dB/MHz), speed of sound (SOS; <1300 or > 1700 m/s), participants with missing data (e.g., quantitative ultrasound, body mass index, dietary data) [30]. Finally, a total of 73,600 participants were included in this study (Fig. 1). All participants provided written informed consent. The study was approved by the Sichuan University Medical Ethical Review Board (K2016038).

### Questionnaire survey

A structured electronic questionnaire was used to collect information on sociodemographic characteristics (age, sex, ethnicity, annual family income, educational attainment, and marital status), lifestyle factors (smoking, alcohol consumption, sleep disorder, and physical activity), and dietary and supplement-related factors (calcium supplement use, vitamin D supplement use and dairy intake).

Educational attainment was categorized into three groups: low, medium, and high. Low education was defined as primary school or below, medium education as junior high school and senior high school, and high education as college or above. Marital status was categorized into two groups: with a spouse (married/cohabiting) and without a spouse (never married, divorced/separated, widowed). Current smokers were defined as individuals who smoked at least one cigarette per day for a continuous period of more than six months; otherwise classified as non-smokers (regardless of tobacco product use). Current alcohol consumers were defined as individuals who drank alcohol at least once per week for a period of more than six months; otherwise classified as non-drinkers (regardless of alcoholic beverage consumption) [31]. Sleep disorder was assessed based on questionnaire responses, including difficulty falling asleep, early awakening, and difficulty maintaining sleep. Physical activity was assessed through a composite score derived from work-related activity, daily commuting mode, routine exercise (type and duration), and routine household chores; higher scores indicated greater physical activity levels. Calcium supplementation and vitamin D supplementation were assessed separately according to whether participants reported using the respective supplement during the previous year. Each variable was independently coded as yes or no [29]. Dairy intake was standardized and modeled per 250 mL.

### Measurement of physiological indices

During the physical examination, participants’ height and weight were measured. To ensure accurate measurements, participants were instructed to remove their hats, remain barefoot, and wear lightweight clothing. BMI was calculated using the formula weight/height² (kg/m²).

### Assessment and classification of QUS-defined bone status

Bone status was assessed at the calcaneus of each participant using a quantitative ultrasound bone densitometer (OSTEOKJ3000, KeJin Inc., Nanjing, China) to obtain the QUI, which is a commonly used bone measure in cohort studies [32, 33]. In brief, bone status was measured by obtaining the speed of sound (SOS, m/s) and broadband ultrasound attenuation (BUA, dB/MHz) values. The QUI was calculated using the formula: QUI = 0.41 × (BUA + SOS) − 571 [34]. The built-in processor also generated T-scores based on a reference database and automatically classified participants into three QUS-defined bone status categories: normal, reduced, and markedly reduced. These categories were treated as device-defined QUS bone status strata and should not be interpreted as DXA-defined clinical diagnoses of osteopenia or osteoporosis.

### Selection and classification of covariates and candidate intermediate variables

**Fig. 2:**
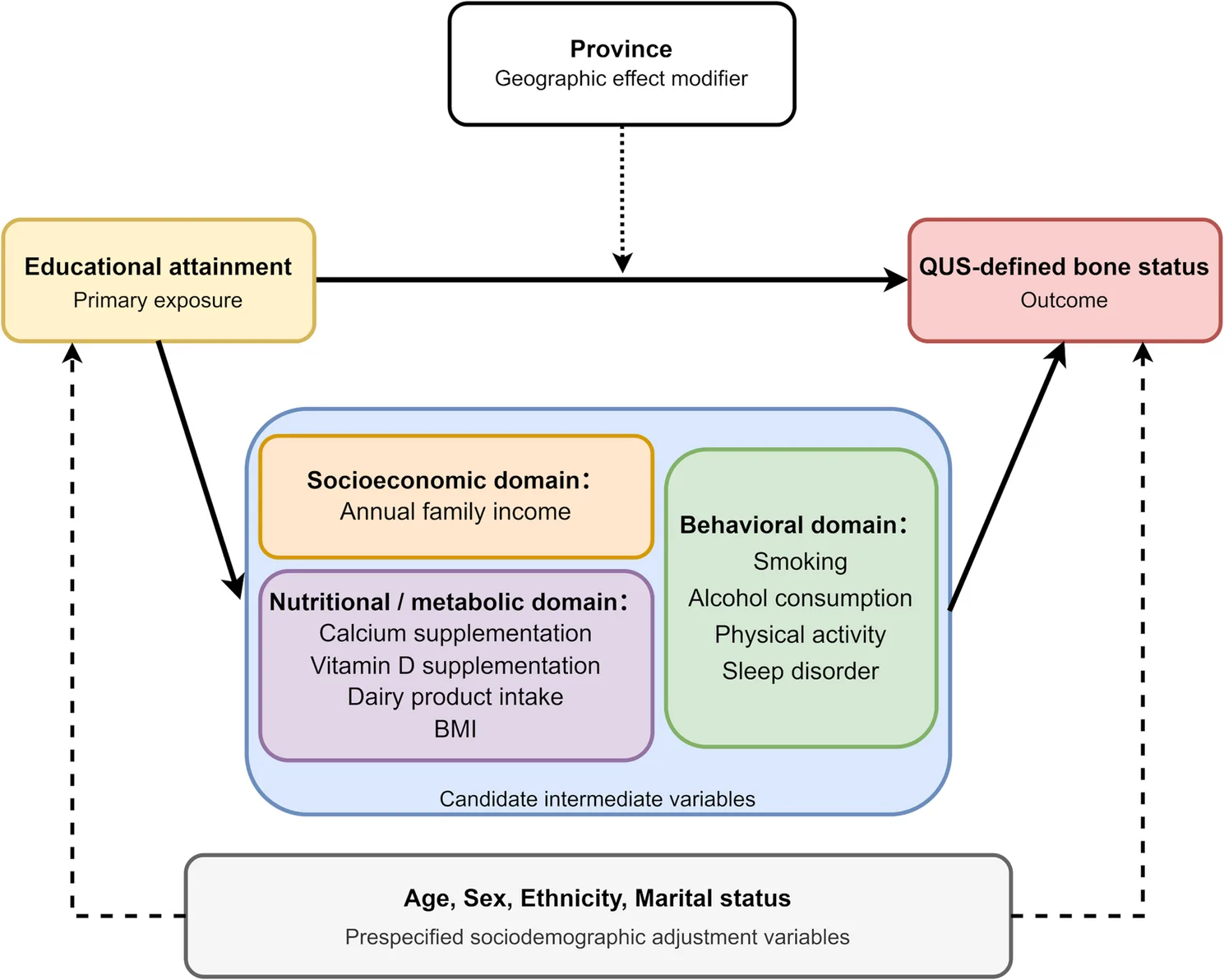
Conceptual framework of province-specific indirect associations linking educational attainment to QUS-defined bone status

Variables were prespecified before model fitting based on previous epidemiological evidence and a hypothesized conceptual framework linking educational attainment with bone status (Fig. 2). Age, sex, ethnicity, and marital status were included as prespecified sociodemographic adjustment variables, with province-specific model specification as appropriate. These variables were retained in the models irrespective of their statistical significance. Province was prespecified as the primary effect modifier because the main objective was to evaluate geographic heterogeneity.

Annual family income, smoking, alcohol consumption, physical activity, sleep disorder, BMI, dairy intake, calcium supplementation, and vitamin D supplementation were considered candidate intermediate variables representing socioeconomic, behavioral, metabolic, and nutritional domains. All prespecified paths involving the candidate intermediate variables were estimated in the province-specific path models irrespective of statistical significance. For visual clarity, only statistically significant paths are displayed in the main diagrams, whereas complete estimates for all prespecified paths, including nonsignificant paths, are provided in Supplementary Results. Statistical significance was used only for graphical presentation and did not determine which paths were estimated. Because all variables were measured at the same baseline examination, their classification as candidate intermediate variables reflected an a priori conceptual hypothesis and should not be interpreted as establishing temporal or causal mediation.

### Statistical analysis

The statistical data were summarized using means (SD) or medians with interquartile ranges (IQR) for the continuous variables and numbers (%) for the categorical variables. Multicollinearity was assessed using adjusted generalized variance inflation factors, expressed as GVIF^1/(2×df)^; values below 2.0 were considered to indicate no substantial multicollinearity. Using the pooled five-province dataset, we assessed effect modification by adding education×selected sociodemographic factors and education× province interaction terms to cumulative logit ordinal regression models fitted with orm (R package rms). Interactions were tested by comparing nested models (with vs. without the interaction term) using a likelihood ratio test based on the change in model deviance; degrees of freedom were defined by the difference in the number of estimated parameters. The education×province interaction was prespecified as the primary test of geographic heterogeneity. Spearman’s rank correlation coefficients were calculated to evaluate pairwise relationships among the study variables, and the results were presented as a correlation matrix. Path models were estimated using weighted least squares mean and variance adjusted (WLSMV), which is appropriate when categorical or ordinal endogenous variables are included. Standardized path coefficients were used to summarize direct, indirect, and total statistical associations among educational attainment, candidate intermediate variables, and bone status. For province-specific path analyses, QUS-defined bone status was reverse-coded as an ordinal score, with higher values representing better bone status (markedly reduced = 0, reduced = 1, and normal = 2). Therefore, positive standardized path coefficients indicate associations with better bone status, whereas negative coefficients indicate associations with poorer bone status. Model fit was evaluated using the root mean square error of approximation (RMSEA), standardized root mean square residual (SRMR), Tucker-Lewis index (TLI), and comparative fit index (CFI). All statistical analyses were performed using the R software (version 4.4.1). A two-tailed *P*-value < 0.05 was considered statistically significant.

### Sensitivity analysis

Several sensitivity analyses were conducted to assess the robustness of the association between educational attainment and QUS-defined bone status. First, sensitivity analyses were conducted in participants from Chongqing, Guizhou, and Yunnan, which represented provinces with intermediate sample sizes. This restriction was used to assess whether the observed associations were robust to the exclusion of provinces with extreme sample sizes. We further adjusted for cardiometabolic and clinical biomarkers, including systolic blood pressure, diastolic blood pressure, fasting glucose, and lipid profile, to evaluate the robustness of the association between educational attainment and bone status.

Second, sex-stratified analyses were performed separately among men and women within each province to account for potential sex differences in the association between educational attainment and bone status. Sex was omitted from the corresponding stratified models, while all other prespecified covariates were retained. In the female-specific models, menopausal status (categorized as premenopausal, perimenopausal, or postmenopausal) was additionally adjusted as a key covariate to evaluate whether the education-bone status association within women was confounded by menopausal status.

Third, to address the potential impact of missing data, we compared the sociodemographic characteristics (age, sex, ethnicity, educational attainment, marital status, and annual family income) of participants excluded due to missing analytical data with those of participants included in the primary complete-case analysis. Between-group differences were assessed using standardized mean differences (SMDs), with an absolute SMD < 0.10 considered negligible, 0.10 to 0.20 considered small, and ≥ 0.20 considered potentially meaningful. Missing analytical values were subsequently handled using multiple imputation by chained equations. Multicollinearity diagnostics and province-specific PPOM analyses were repeated across the imputed datasets to assess whether missing data materially influenced the study conclusions.

For all sensitivity analyses described above, we first considered proportional odds (PO) ordinal logistic regression and assessed the parallel-lines assumption using the global Brant test. When the assumption was satisfied (*P* ≥ 0.05), PO models were applied; otherwise, partial proportional odds models (PPOMs; generalized ordered logit models) were used, in which the proportionality constraint was relaxed only for variables (or, for multicategorical predictors, the variable as a whole) that violated the assumption. For ordinal regression sensitivity analyses, QUS-defined bone status was coded according to increasing severity of bone status reduction (normal = 0, reduced = 1, and markedly reduced = 2). Accordingly, ORs > 1 indicate higher odds of being classified into a more reduced bone-status category, whereas ORs < 1 indicate lower odds.

## Results

### Characteristics of study population

A total of 73,600 participants were included, with a mean age of 51.54 years (SD, 11.43); 45,681 (62.1%) were women. Overall, 44,600 (60.6%), 25,794 (35.0%), and 3,206 (4.4%) participants were classified into the normal, reduced, and markedly reduced QUS-defined bone status categories, respectively. Low, medium, and high educational attainment accounted for 53.45%, 36.24%, and 10.31% of the study population. The distributions of most participant characteristics differed across the three bone-status categories, whereas vitamin D supplementation did not differ significantly (*P* = 0.087) (Table 1).

**Table 1:** General characteristics of the study participants according to QUS-defined bone status

Variable	Overall *N* = 73,600 ^1^	Normal *N* = 44,600 ^1^	Reduced *N* = 25,794 ^1^	Markedly reduced *N* = 3,206 ^1^	*P* ^2^
Sex					< 0.001
Female	45,681 (62.07%)	31,965 (71.67%)	12,598 (48.84%)	1,118 (34.87%)	
Male	27,919 (37.93%)	12,635 (28.33%)	13,196 (51.16%)	2,088 (65.13%)	
Age (years)	51.54 ± 11.43	49.11 ± 10.48	54.99 ± 11.73	57.53 ± 12.16	< 0.001
Age group (years)					< 0.001
≤ 45	23,325 (31.69%)	16,873 (37.83%)	5,859 (22.71%)	593 (18.50%)	
45–65	40,167 (54.57%)	24,145 (54.14%)	14,388 (55.78%)	1,634 (50.97%)	
> 65	10,108 (13.73%)	3,582 (8.03%)	5,547 (21.51%)	979 (30.54%)	
BMI (kg/m^2^)					< 0.001
≤ 24	37,380 (50.79%)	22,409 (50.24%)	13,594 (52.70%)	1,377 (42.95%)	
24–28	26,807 (36.42%)	16,561 (37.13%)	9,094 (35.26%)	1,152 (35.93%)	
≥ 28	9,413 (12.79%)	5,630 (12.62%)	3,106 (12.04%)	677 (21.12%)	
Marital status					< 0.001
Single	7,980 (10.84%)	4,093 (9.18%)	3,397 (13.17%)	490 (15.28%)	
Married	65,620 (89.16%)	40,507 (90.82%)	22,397 (86.83%)	2,716 (84.72%)	
Ethnicity					< 0.001
Han Chinese	41,113 (55.86%)	24,178 (54.21%)	15,210 (58.97%)	1,725 (53.81%)	
Ethnic Minorities	32,487 (44.14%)	20,422 (45.79%)	10,584 (41.03%)	1,481 (46.19%)	
Annual family income (CNY/year)					< 0.001
< 60,000	53,464 (72.64%)	32,438 (72.73%)	18,578 (72.02%)	2,448 (76.36%)	
≥ 60,000	20,136 (27.36%)	12,162 (27.27%)	7,216 (27.98%)	758 (23.64%)	
Educational attainment					< 0.001
Low	39,341 (53.45%)	23,730 (53.21%)	13,670 (53.00%)	1,941 (60.54%)	
Medium	26,674 (36.24%)	15,940 (35.74%)	9,708 (37.64%)	1,026 (32.00%)	
High	7,585 (10.31%)	4,930 (11.05%)	2,416 (9.37%)	239 (7.45%)	
Smoking status					< 0.001
No	59,164 (80.39%)	38,170 (85.58%)	18,798 (72.88%)	2,196 (68.50%)	
Yes	14,436 (19.61%)	6,430 (14.42%)	6,996 (27.12%)	1,010 (31.50%)	
Alcohol consumption					< 0.001
No	43,261 (58.78%)	27,207 (61.00%)	14,191 (55.02%)	1,863 (58.11%)	
Yes	30,339 (41.22%)	17,393 (39.00%)	11,603 (44.98%)	1,343 (41.89%)	
Sleep disorder					< 0.001
No	41,876 (56.90%)	26,040 (58.39%)	14,152 (54.87%)	1,684 (52.53%)	
Yes	31,724 (43.10%)	18,560 (41.61%)	11,642 (45.13%)	1,522 (47.47%)	
Physical activity					< 0.001
Low	18,400 (25.00%)	9,566 (21.45%)	7,606 (29.49%)	1,228 (38.30%)	
Medium	36,798 (50.00%)	22,744 (51.00%)	12,644 (49.02%)	1,410 (43.98%)	
High	18,402 (25.00%)	12,290 (27.56%)	5,544 (21.49%)	568 (17.72%)	
Province					< 0.001
Yunnan	19,145 (26.01%)	12,144 (27.23%)	6,453 (25.02%)	548 (17.09%)	
Guizhou	15,190 (20.64%)	9,473 (21.24%)	5,275 (20.45%)	442 (13.79%)	
Sichuan	21,042 (28.59%)	13,093 (29.36%)	7,121 (27.61%)	828 (25.83%)	
Chongqing	14,421 (19.59%)	7,413 (16.62%)	5,891 (22.84%)	1,117 (34.84%)	
Tibet	3,802 (5.17%)	2,477 (5.55%)	1,054 (4.09%)	271 (8.45%)	
Calcium supplementation					< 0.001
No	64,634 (87.82%)	39,247 (88.00%)	22,517 (87.30%)	2,870 (89.52%)	
Yes	8,966 (12.18%)	5,353 (12.00%)	3,277 (12.70%)	336 (10.48%)	
Vitamin D supplementation					0.087
No	72,193 (98.09%)	43,708 (98.00%)	25,339 (98.24%)	3,146 (98.13%)	
Yes	1,407 (1.91%)	892 (2.00%)	455 (1.76%)	60 (1.87%)	
Dairy product intake (/250mL)	1.47 ± 2.31	1.48 ± 2.30	1.45 ± 2.31	1.43 ± 2.32	< 0.001

^1^Mean ± SD or Frequency (%)

^2^Pearson’s Chi-squared test or Kruskal-Wallis rank sum test

### Effect modification and geographic heterogeneity

Model diagnostics indicated no substantial multicollinearity in the pooled effect-modification model, with all adjusted GVIF values below 2.0. The education×province interaction was statistically significant (χ² = 67.387, df = 8, *P* < 0.001), supporting geographic heterogeneity in the education-bone status association (Table 2).

**Table 2:** Interaction tests of educational attainment with selected sociodemographic factors and province

Interaction term	Chi-square (χ²)	df	*P* value
Educational attainment × Sex	168.747	2	< 0.001
Educational attainment × Age	207.681	4	< 0.001
Educational attainment × Ethnicity	20.941	2	< 0.001
Educational attainment × Marital status	21.680	2	< 0.001
Educational attainment × Province	67.387	8	**< **0.001

### Correlation matrix of the variables

To conduct the path analysis, we first tested the correlations of the variables across the five provinces, with the results presented in Supplementary Table S1.

### Province-specific path analysis

Model-fit indices for the five province-specific path models were as follows: Sichuan (CFI = 0.999, TLI = 0.998, RMSEA = 0.015, SRMR = 0.017), Chongqing (CFI = 0.987, TLI = 0.954, RMSEA = 0.009, SRMR = 0.009), Guizhou (CFI = 0.975, TLI = 0.936, RMSEA = 0.009, SRMR = 0.016), Yunnan (CFI = 0.998, TLI = 0.996, RMSEA = 0.021, SRMR = 0.026), and Tibet (CFI = 0.968, TLI = 0.911, RMSEA = 0.009, SRMR = 0.024). Except for Yunnan, educational attainment showed significant total and direct associations with QUS-defined bone status in the other four provinces. Specifically, in Sichuan, Guizhou, and Tibet, higher educational attainment was significantly associated with poorer bone status (*P <* 0.05). In contrast, in Chongqing, higher educational attainment was significantly associated with better bone status (*P <* 0.05). Further analysis revealed that, except for Tibet, other provinces had multiple distinct statistically significant indirect associations linking educational attainment to bone status. For visual clarity, only statistically significant paths are displayed; complete estimates for all prespecified paths, including nonsignificant estimates, are provided in Supplementary Table S2.

### Path analysis of sichuan participants

**Fig. 3:**
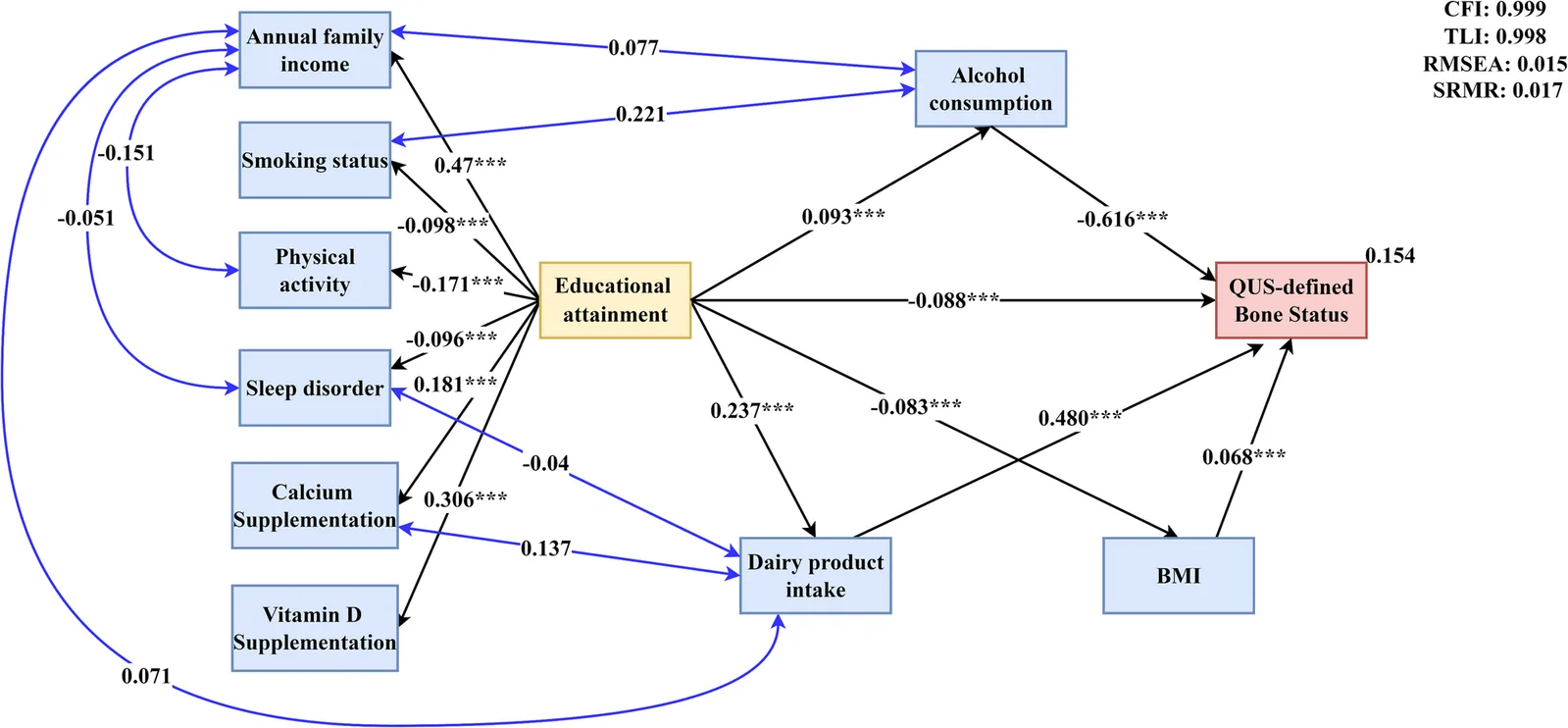
Results of path analysis for Sichuan province, with standardized coefficients presented. The yellow box represents the primary exposure (educational attainment), the red box represents the outcome (QUS-defined bone status), and the blue boxes represent candidate intermediate variables. Black single-headed arrows indicate standardized regression path coefficients, whereas blue curved double-headed arrows indicate covariance and do not imply directional or causal effects. Positive standardized coefficients indicate associations with better bone status, whereas negative coefficients indicate associations with poorer bone status. *P < 0.05, **P < 0.01, ***P < 0.001

In Sichuan (Fig. 3), educational attainment was associated with bone status through potential indirect associations involving alcohol consumption, dairy product intake, and BMI. Among these indirect associations, the largest inverse estimate involved alcohol consumption: higher educational attainment was associated with greater alcohol consumption (β = 0.093, *P <* 0.001), and greater alcohol consumption was associated with poorer bone status (β = -0.616, *P <* 0.001). Dairy product intake was the largest positive indirect association, with higher educational attainment associated with higher dairy intake (β = 0.237, *P <* 0.001), which positively correlated with bone status (β = 0.480, *P <* 0.001).

### Path analysis of chongqing participants

**Fig. 4:**
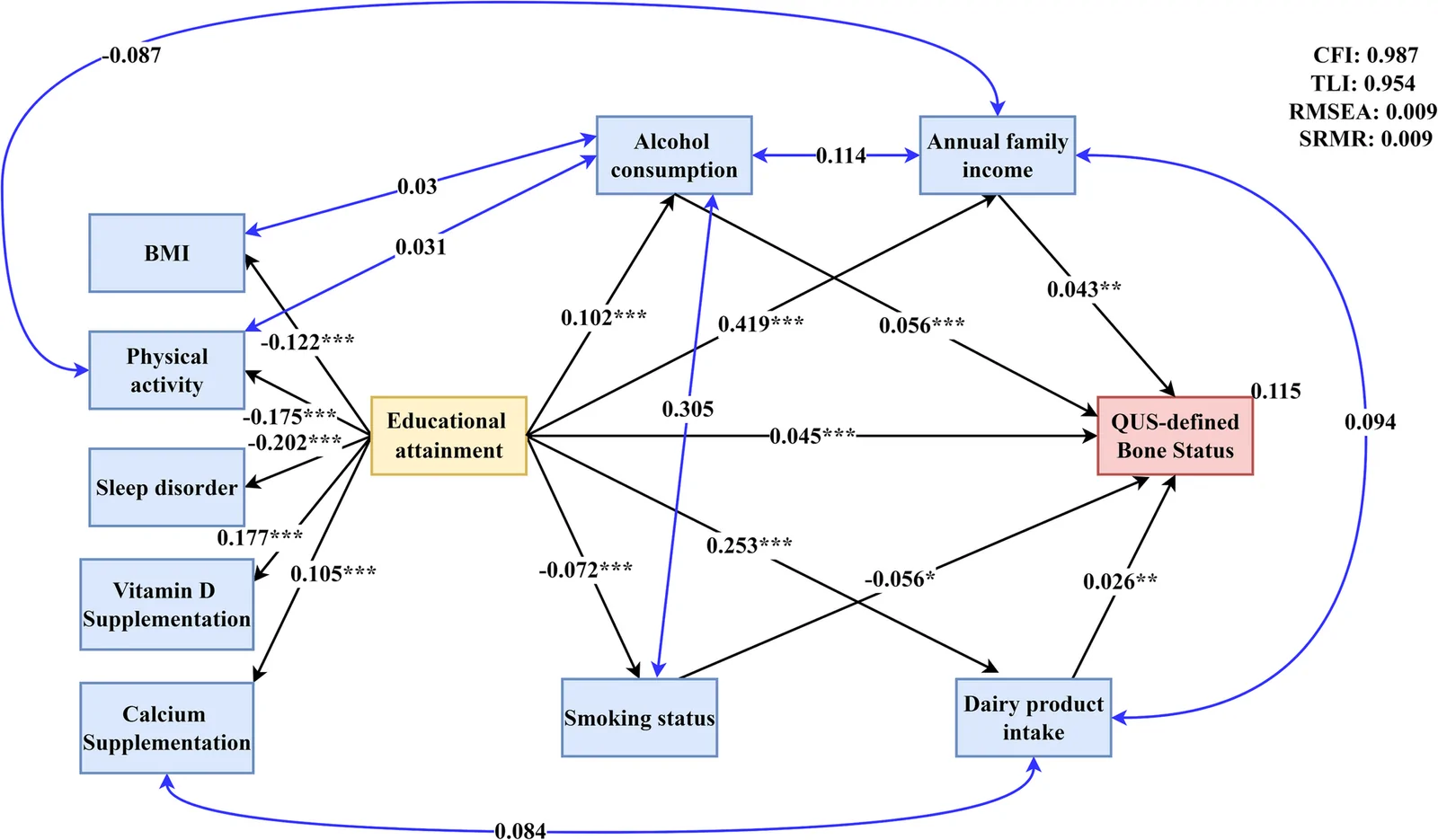
Results of path analysis for Chongqing province, with standardized coefficients presented. The yellow box represents the primary exposure (educational attainment), the red box represents the outcome (QUS-defined bone status), and the blue boxes represent candidate intermediate variables. Black single-headed arrows indicate standardized regression path coefficients, whereas blue curved double-headed arrows indicate covariance and do not imply directional or causal effects. Positive standardized coefficients indicate associations with better bone status, whereas negative coefficients indicate associations with poorer bone status. *P < 0.05, **P < 0.01, ***P < 0.001

In Chongqing (Fig. 4), the largest positive indirect association involved annual family income, followed by dairy product intake. Higher educational attainment was associated with higher annual family income (β = 0.419, *P <* 0.001), and annual family income was positively correlated with bone status (β = 0.043, *P* = 0.002). Smoking is widely recognized as a risk factor for poorer bone outcomes. In Chongqing, the analysis revealed that higher educational attainment was associated with lower smoking rates (β = -0.072, *P <* 0.001), and smoking was negatively correlated with bone status (β = -0.056, *P* = 0.031).

### Path analysis of guizhou participants

**Fig. 5:**
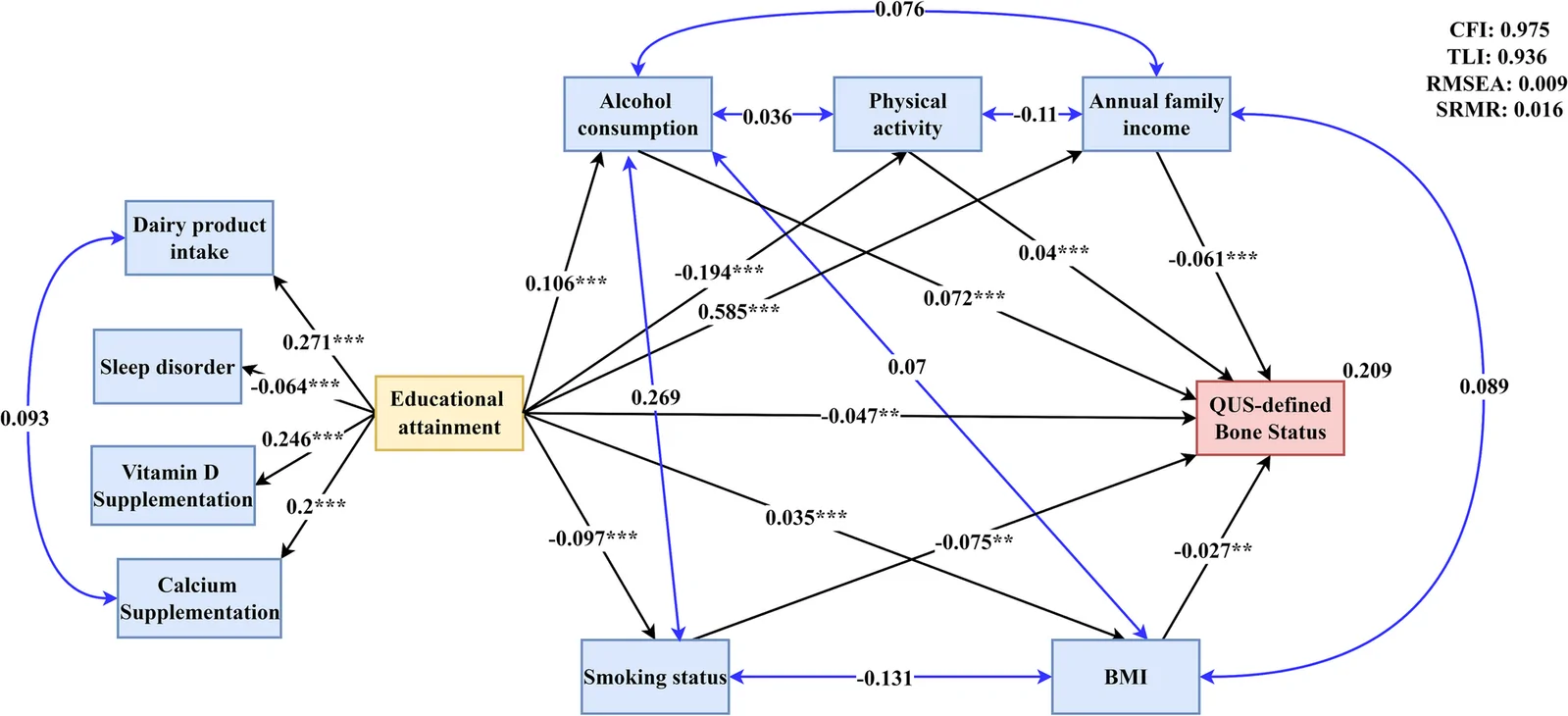
Results of path analysis for Guizhou province, with standardized coefficients presented. The yellow box represents the primary exposure (educational attainment), the red box represents the outcome (QUS-defined bone status), and the blue boxes represent candidate intermediate variables. Black single-headed arrows indicate standardized regression path coefficients, whereas blue curved double-headed arrows indicate covariance and do not imply directional or causal effects. Positive standardized coefficients indicate associations with better bone status, whereas negative coefficients indicate associations with poorer bone status. *P < 0.05, **P < 0.01, ***P < 0.001

In Guizhou (Fig. 5), higher educational attainment was associated with lower levels of physical activity (β = -0.194, *P <* 0.001), and physical activity was positively correlated with bone status (β = 0.04, *P <* 0.001). Unlike in other provinces, although higher educational attainment was associated with higher annual family income (β = 0.585, *P <* 0.001), annual family income in Guizhou showed a negative correlation with bone status (β = -0.061, *P <* 0.001).

### Path analysis of Yunnan participants

**Fig. 6:**
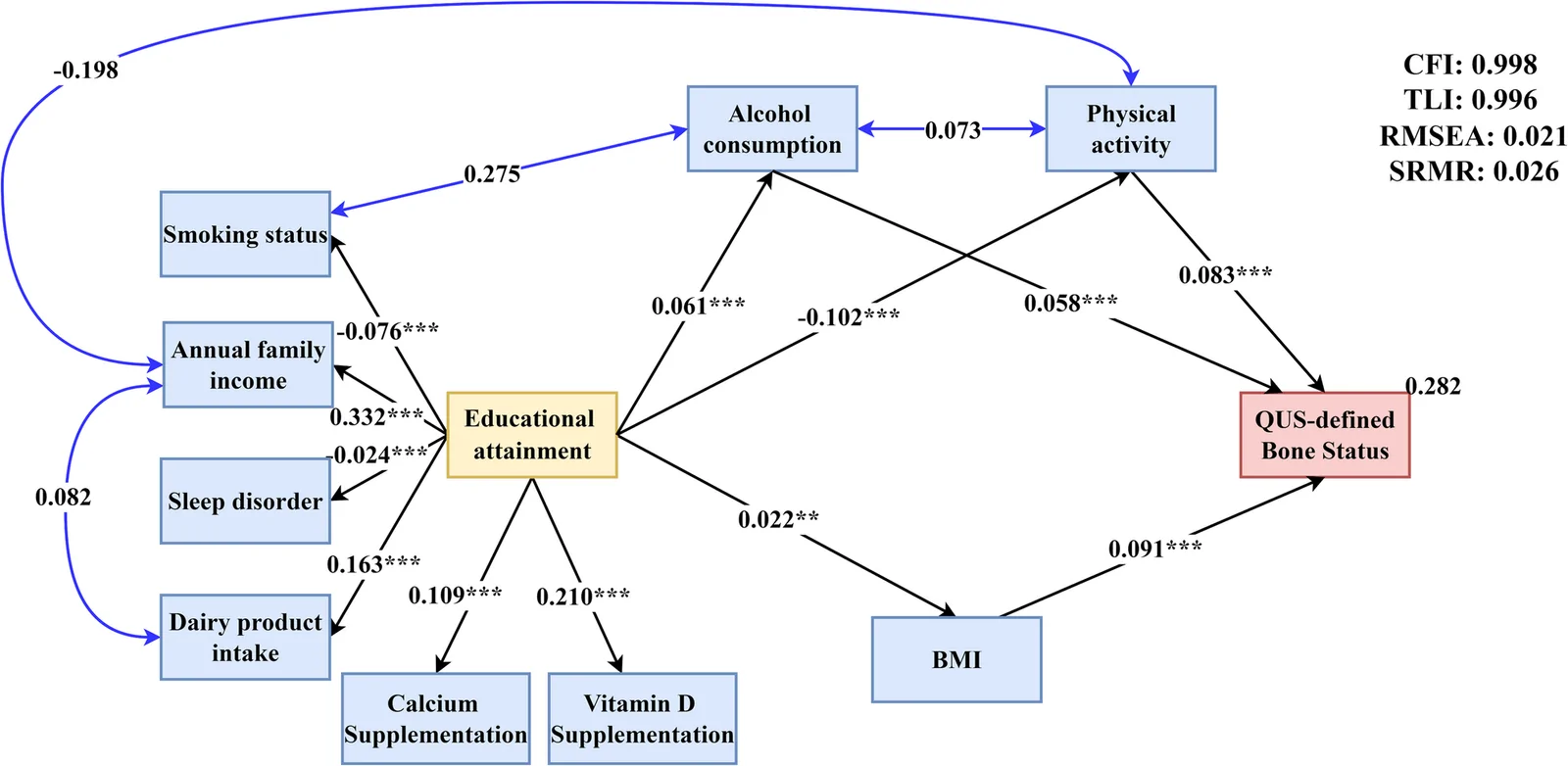
Results of path analysis for Yunnan province, with standardized coefficients presented. The yellow box represents the primary exposure (educational attainment), the red box represents the outcome (QUS-defined bone status), and the blue boxes represent candidate intermediate variables. Black single-headed arrows indicate standardized regression path coefficients, whereas blue curved double-headed arrows indicate covariance and do not imply directional or causal effects. Positive standardized coefficients indicate associations with better bone status, whereas negative coefficients indicate associations with poorer bone status. *P < 0.05, **P < 0.01, ***P < 0.001

Although educational attainment did not show significant total or direct effects on bone status in Yunnan, statistically significant indirect associations were observed (Fig. 6). Similar to Guizhou, higher educational attainment was associated with lower levels of physical activity (β = -0.102, *P <* 0.001), and physical activity was positively correlated with bone status (β = 0.083, *P <* 0.001). Additionally, higher educational attainment was associated with higher BMI (β = 0.022, *P <* 0.001), and BMI was positively correlated with bone status (β = 0.091, *P <* 0.001).

### Sensitivity analysis

Sensitivity analyses were broadly consistent with the primary province-specific findings. After additional adjustment for systolic blood pressure (SBP), diastolic blood pressure (DBP), fasting glucose, and lipid markers in the three provinces with intermediate sample sizes (Chongqing, Guizhou, and Yunnan), higher educational attainment remained associated with better bone status in Chongqing and poorer bone status in Guizhou, whereas no statistically significant association was observed in Yunnan. No substantial multicollinearity was detected in these expanded models, with all adjusted GVIF values below 2.0. The global Brant tests were significant in all three models (all *P* < 0.001). Educational attainment required threshold-specific coefficients in Chongqing but retained common coefficients in Guizhou and Yunnan; detailed Brant-test results and PPOM specifications are provided in Supplementary Table S4 (Supplementary Tables S3-S7).

Sex-stratified analyses showed broadly similar geographic heterogeneity, although the magnitude and statistical significance of the estimates varied by sex, province, and educational-attainment category. Among women, additional adjustment for menopausal status did not materially change the overall pattern. In Tibet, the sex-stratified estimates were no longer statistically significant and showed wide confidence intervals, consistent with reduced precision after stratification of the smallest provincial sample; nevertheless, most ORs remained above 1.0. The global Brant tests rejected the proportional-odds assumption in all five female-specific models and in the male-specific models for Sichuan, Chongqing, Guizhou, and Yunnan, but not among Tibetan men (*P* = 0.0649). Educational attainment required threshold-specific coefficients in Chongqing and Tibet among women, whereas common coefficients were retained in the other female-specific models and in all male-specific models (Supplementary Tables S4, S8-9).

Participants excluded because of missing data (13.0%) showed some differences from the analytic sample, particularly in educational attainment (SMD = 0.205), ethnicity (SMD = 0.193), and annual family income (SMD = 0.180), whereas differences in age (SMD = 0.067) and sex (SMD = 0.108) were smaller (Supplementary Table S10). Multiple imputation was conducted as a separate sensitivity analysis to evaluate the potential influence of missing data. All adjusted GVIF values remained below 2.0, indicating no substantial multicollinearity (Supplementary Table S11). The imputed analyses broadly reproduced the main geographic pattern, including the positive association in Chongqing, the inverse association in Sichuan, Guizhou and Tibet, and the null association in Yunnan (Supplementary Table S12). The global Brant tests rejected the proportional-odds assumption in all five imputed province-specific models. Educational attainment required threshold-specific coefficients in Chongqing and Tibet but retained common coefficients in Sichuan, Guizhou, and Yunnan. Supplementary Table S4 summarizes the Brant-test results for each sensitivity analysis, including the specific predictor terms that violated the proportional-odds assumption and the variables for which the proportionality constraint was subsequently relaxed.

## Discussion

Based on large-scale cross-sectional data from five provinces in Southwest China, this study showed that the association between educational attainment and QUS-defined bone status is strongly context dependent, rather than uniformly positive. This heterogeneity suggests that the association between educational attainment and bone status may vary according to local socioeconomic conditions, occupational patterns, lifestyle behaviors, and access to health-related resources. Notably, the association varied substantially even within a single country. Specifically, Southwest China illustrates this well: the region is marked by pronounced internal heterogeneity in socioeconomic development, physical geography, cultural practices, and access to health resources [35, 36]. In several provinces, higher educational attainment was associated with poorer bone status, challenging the conventional assumption that “higher education is consistently associated with better health”. This pattern is not without precedent: Norwegian researchers have predicted that, by 2050, the incidence of hip fracture in Norway will double, with most cases expected to occur in people with secondary and tertiary education [37].

Lifestyle factors (alcohol consumption, smoking, and physical activity) were implicated in province-specific indirect statistical associations between educational attainment and bone status, though their direction and magnitude varied geographically. In Sichuan, the strongest inverse indirect association involved alcohol consumption, consistent with its high prevalence of at least weekly drinking (50.2%), heavy average weekly alcohol intake (414.6 g/week), and a particularly high proportion consuming strong spirits [38]. Conversely, alcohol consumption showed a positive indirect statistical association with bone status in Chongqing, Guizhou, and Yunnan. This regional divergence may reflect a nonlinear J- or U-shaped dose-response relationship, whereby light to moderate alcohol consumption is associated with better bone status, whereas long-term heavy drinking is detrimental to skeletal health; the positive association between alcohol intake and BMI may also partly explain this pattern [39–43]. Similarly, individuals with higher educational attainment are more likely to consume alcohol than are those with lower levels of education [44, 45]. Differences in alcohol types, drinking frequency, and consumption levels across provinces may partly explain these opposing directions of association.

Chongqing, the most urbanized and economically developed of the five southwestern regions, is characterized by a more balanced distribution of health-care resources. Higher educational attainment in Chongqing was linked to greater access to more health-promoting behaviors, including reduced smoking (a well-established osteoporosis risk factor [46, 47]) and increased dairy intake; a similar positive indirect statistical association involving dairy consumption was also observed in Sichuan. In Guizhou and Yunnan, by contrast, the most prominent indirect statistical association involved physical activity: higher educational attainment was associated with lower physical activity, corresponding to an inverse indirect statistical association with bone status. Similarly, higher annual family income was also inversely associated with physical activity in Guizhou, which may be consistent with the greater likelihood that higher-income and better-educated individuals engage in sedentary occupations. This is consistent with substantial evidence that regular and proper exercise is associated with improved skeletal outcomes through the intricate crosstalk between osteoblasts and osteoclasts [48–50], and suggests that reduced physical activity may become an increasingly common risk behavior among highly educated populations as lifestyles grow more sedentary [16, 23]. Together, these patterns indicate that the public health meaning of education is not fixed, but depends on how it intersects with local labor patterns, nutritional and economic conditions, and everyday behaviors.

BMI played a dual role across different regional contexts, and its association with bone status was not a simple linear positive relationship. In our study, BMI showed a positive indirect association in Yunnan but showed a negative indirect association in Sichuan and Guizhou. This divergence may be consistent with the hypothesis of a potential inverted U-shaped relationship between BMI and bone status [51]: moderate weight gain may enhance bone formation through mechanical loading, whereas once a threshold is exceeded, subsequent metabolic abnormalities (such as insulin resistance and chronic low-grade inflammation) may compromise skeletal structure [42, 52]. Notably, in high-altitude areas, studies have revealed a greater prevalence of osteoporosis among individuals with a BMI greater than 28 kg/m², suggesting that under specific environmental stresses [53], the “beneficial window” of BMI for bone health may be narrower and may even reverse beyond a certain point.

In the context of Tibet’s unique high-altitude, low atmospheric pressure, oxygen deficiency environment, traditional dietary patterns (e.g., diets dominated by barley and Tibetan noodles), and physical activity patterns, variations in bone status may be more directly shaped by physiological adaptation mechanisms and strong environmental selection pressures [54]. Accordingly, in ecological settings such as Tibet, conventional socioeconomic and behavioral indicators may be insufficient to fully explain variation in bone status, highlighting the need for more locally grounded public health research.

Calcaneal QUS is practical for large-scale field studies because it is portable, radiation-free, and operationally feasible, although its measurements are related to, rather than interchangeable with, DXA-derived BMD. Previous studies have shown that heel QUS reflects calcaneal mechanical properties and is significantly, but incompletely, correlated with DXA measurements at central skeletal sites [55, 56]. In a related analysis of 65,906 participants from the same China Multi-Ethnic Cohort, Yan et al. used the same OSTEOKJ3000 device and noted, based on previous validation studies, that heel QUS measurements correlate closely with DXA-measured BMD at the heel and moderately with DXA-measured BMD at the spine and hip [57]. In Chinese adults, a QUS T-score threshold of − 1.8 yielded 73.2% sensitivity and 92.3% specificity for DXA-defined osteoporosis, whereas a threshold of − 2.35 increased specificity to 100% at the cost of lower sensitivity [58]. These findings support the operational feasibility of QUS in multi-ethnic populations in Southwest China and its utility for large-scale population screening and risk stratification. Consistently, the International Society for Clinical Densitometry (ISCD) recognizes the heel as the validated site for clinical QUS and notes that validated heel QUS devices predict fragility fractures independently of central DXA BMD [59]. Nevertheless, central DXA remains preferred for diagnosis and therapeutic decision-making.

From a public health perspective, our findings suggest that educational attainment should not be assumed to have a uniform association with QUS-defined bone status across regions. Prevention strategies may therefore benefit from greater attention to locally relevant behavioral and occupational factors, particularly physical inactivity, smoking, alcohol consumption, and dietary patterns. Highly educated populations should not automatically be regarded as a low-risk group, especially in regions where sedentary work and unfavorable lifestyle behaviors may offset the potential health advantages associated with education. These findings support region-specific approaches to identifying and addressing bone-health risks in Southwest China.

### Limitations

Our study has several limitations. First, bone status was assessed using calcaneal QUS rather than central DXA because many study sites were located in remote or high-altitude areas. Although suitable for large-scale field studies, QUS measurements are not interchangeable with DXA-derived BMD. Second, the cross-sectional design precludes temporal or causal inference; therefore, the path analyses should be interpreted as statistical associations rather than established causal pathways. Third, diet, alcohol consumption, smoking, and physical activity were assessed using self-reported questionnaires and may be subject to recall or reporting bias. Fourth, individual-level environmental and biological metrics were not fully quantified. Regional ultraviolet radiation data could not accurately capture personal exposure and were therefore not included, and sex hormone concentrations were not measured, although menopausal status was adjusted for in female-specific sensitivity analyses. Fifth, participants were recruited from selected settlements and communities using multistage stratified cluster sampling. Despite broad geographic and ethnic coverage, the findings may not be fully generalizable to all populations within or beyond Southwest China. Finally, 13.0% of eligible participants were excluded because of missing analytical data, and the excluded participants differed from the analytic sample particularly in educational attainment, ethnicity, and annual family income. Because educational attainment was the primary exposure and ethnicity and income may be related to regional socioeconomic context, complete-case exclusion may have introduced some degree of selection bias. Although multiple-imputation analyses yielded broadly consistent province-specific findings, residual bias related to missing data cannot be excluded.

## Conclusion

In this large multi-provincial study from Southwest China, educational attainment was associated with QUS-defined bone status in a province-specific rather than uniform manner. Province-specific indirect statistical associations involving behavioral, nutritional, and socioeconomic factors were observed, but the cross-sectional design does not permit causal interpretation. The main findings were broadly consistent across sensitivity analyses and require confirmation in longitudinal studies using DXA-based assessment.

## Supplementary Information

Supplementary Material 1.

## Data Availability

The datasets used and/or analysed during the current study are not publicly available due to participant privacy but are available from the corresponding author on reasonable request.

## Abbreviations

BMI: Body mass index
BUA: Broadband ultrasound attenuation
CFI: Comparative fit index
CMEC: China Multi-Ethnic Cohort
DBP: Diastolic blood pressure
DXA: Dual-energy X-ray absorptiometry
IQR: Interquartile range
PO: Proportional odds
PPOM: Partial proportional odds model
QUS: Quantitative ultrasound
QUI: Quantitative ultrasound index
RMSEA: Root mean square error of approximation
SBP: Systolic blood pressure
SD: Standard deviation
SES: Socioeconomic status
SOS: Speed of sound
SRMR: Standardized root mean square residual
TLI: Tucker‒Lewis index
WLSMV: Weighted least squares mean and variance adjusted
