# Supplementary material for: Geographic heterogeneity in the association between educational attainment and QUS-defined bone status among adults in Southwest China: a cross-sectional study
Source: BMC Public Health. 2026 Aug 27;26:3143. doi: 10.1186/s12889-026-29217-9 (PMC13721239; doi:10.1186/s12889-026-29217-9)
Supplement: Supplementary file 1 [file 12889_2026_29217_MOESM1_ESM.zip › Revised Supplementary Material/Revised Supplementary Results.pdf]

## Supplementary Results

### Supplementary Information Summary

Table S2. Complete standardized associations among educational attainment, candidate intermediate variables, and QUS-defined bone status across provinces .................................. 2

Table S3. Multicollinearity diagnostics for all variables in the province-specific models ........ 3

Table S4. Summary of Brant-test diagnostics and PPOM specification in sensitivity analyses 4

Table S5. Educational attainment and bone status in Chongqing: PPOM sensitivity analysis .. 6

Table S6. Educational attainment and bone status in Guizhou: PPOM sensitivity analysis ...... 8

Table S7. Educational attainment and bone status in Yunnan: PPOM sensitivity analysis ....... 9

Table S8. Province-specific educational attainment-bone status associations among women: PPOM sensitivity analysis ........................................................................................ 11

Table S9. Province-specific educational attainment-bone status associations among men: sensitivity analysis ................................................................................................ 12

Table S10. Selected sociodemographic characteristics of analytic participants and participants excluded for missing data ........................................................................................ 13

Table S11. Multicollinearity diagnostics for province-specific models after multiple imputation ........................................................................................................ 14

Table S12. Province-specific educational attainment-bone status associations after multiple imputation: PPOM sensitivity analysis ........................................................................ 15

**Note：**

**Supplementary Table S1. Correlation matrix of study variables across the five provinces(XLSX).** This table is provided as a separate Excel file and is not reproduced in this document.

**Table S2.** Complete standardized associations among educational attainment, candidate intermediate variables, and QUS-defined bone status across provinces

| Province | Candidate intermediate variable | Education → Candidate β | *P* | Candidate → Bone status β | *P* | Education → Bone status β | *P* |
|---|---|---|---|---|---|---|---|
| Sichuan | Annual family income | 0.47 | <0.001 | -0.011 | 0.542 | -0.088 | <0.001 |
| | Smoking | -0.098 | <0.001 | 0.135 | 0.152 | | |
| | **Alcohol consumption** | **0.093** | **<0.001** | **-0.616** | **<0.001** | | |
| | Physical activity | -0.171 | <0.001 | -0.036 | 0.579 | | |
| | Sleep disorder | -0.096 | <0.001 | -0.002 | 0.879 | | |
| | Calcium supplementation | 0.181 | <0.001 | -0.039 | 0.085 | | |
| | Vitamin D supplementation | 0.306 | <0.001 | -0.042 | 0.057 | | |
| | **Dairy product intake** | **0.237** | **<0.001** | **0.48** | **<0.001** | | |
| | **BMI** | **-0.083** | **<0.001** | **0.068** | **<0.001** | | |
| Chongqing | **Annual family income** | **0.419** | **<0.001** | **0.043** | **0.002** | 0.045 | <0.001 |
| | **Smoking** | **-0.072** | **<0.001** | **-0.056** | **0.031** | | |
| | **Alcohol consumption** | **0.102** | **<0.001** | **0.056** | **<0.001** | | |
| | Physical activity | -0.175 | <0.001 | 0.013 | 0.198 | | |
| | Sleep disorder | -0.202 | <0.001 | -0.024 | 0.063 | | |
| | Calcium supplementation | 0.105 | <0.001 | -0.006 | 0.755 | | |
| | Vitamin D supplementation | 0.177 | <0.001 | 0.088 | 0.129 | | |
| | **Dairy product intake** | **0.253** | **<0.001** | **0.026** | **0.009** | | |
| | BMI | -0.122 | <0.001 | 0.014 | 0.117 | | |
| Guizhou | **Annual family income** | **0.585** | **<0.001** | **-0.061** | **<0.001** | -0.047 | 0.002 |
| | **Smoking** | **-0.097** | **<0.001** | **-0.075** | **0.009** | | |
| | **Alcohol consumption** | **0.106** | **<0.001** | **0.072** | **<0.001** | | |
| | **Physical activity** | **-0.194** | **<0.001** | **0.04** | **<0.001** | | |
| | Sleep disorder | -0.064 | <0.001 | -0.02 | 0.087 | | |
| | Calcium supplementation | 0.2 | <0.001 | 0.016 | 0.336 | | |
| | Vitamin D supplementation | 0.246 | <0.001 | 0.022 | 0.747 | | |
| | Dairy product intake | 0.271 | <0.001 | 0.012 | 0.216 | | |
| | **BMI** | **0.035** | **<0.001** | **-0.027** | **0.003** | | |
| Yunnan | Annual family income | 0.332 | <0.001 | -0.023 | 0.108 | 0.015 | 0.155 |
| | Smoking | -0.076 | <0.001 | -0.013 | 0.633 | | |
| | **Alcohol consumption** | **0.061** | **<0.001** | **0.058** | **<0.001** | | |
| | **Physical activity** | **-0.102** | **<0.001** | **0.083** | **<0.001** | | |

| Province | Candidate intermediate variable | Education → Candidate β | *P* | Candidate → Bone status β | *P* | Education → Bone status β | *P* |
|---|---|---|---|---|---|---|---|
| | Sleep disorder | -0.024 | 0.018 | -0.001 | 0.957 | | |
| | Calcium supplementation | 0.109 | <0.001 | -0.020 | 0.545 | | |
| | Vitamin D supplementation | 0.210 | <0.001 | -0.030 | 0.497 | | |
| | Dairy product intake | 0.163 | <0.001 | -0.009 | 0.529 | | |
| | **BMI** | **0.022** | **0.009** | **0.091** | **<0.001** | | |
| Tibet | Annual family income | 0.277 | <0.001 | 0.023 | 0.443 | -0.067 | 0.012 |
| | Smoking | -0.02 | 0.377 | -0.092 | 0.015 | | |
| | Alcohol consumption | 0.043 | 0.047 | 0.032 | 0.239 | | |
| | Physical activity | -0.012 | 0.515 | 0.041 | 0.027 | | |
| | Sleep disorder | 0.051 | 0.015 | -0.017 | 0.491 | | |
| | Calcium supplementation | 0.271 | <0.001 | 0.023 | 0.688 | | |
| | Vitamin D supplementation | 0.199 | <0.001 | -0.166 | 0.345 | | |
| | Dairy product intake | 0.023 | 0.113 | 0.006 | 0.764 | | |
| | BMI | 0.008 | 0.597 | -0.019 | 0.288 | | |

Notes: All prespecified candidate intermediate variables are shown irrespective of statistical significance. Bone status refers to QUS-defined bone status, and all β coefficients are standardized estimates. "Education → Candidate," "Candidate → Bone status," and "Education → Bone status" denote the corresponding model-estimated statistical associations. Models were adjusted for age, sex, ethnicity (where estimable), and marital status. Arrows indicate model specification only and do not imply temporal ordering, mediation, or causality. Positive standardized coefficients indicate associations with better bone status, whereas negative coefficients indicate associations with poorer bone status.

**Table S3.** Multicollinearity diagnostics for all variables in the province-specific models

| Variable | Sichuan | Chongqing | Guizhou | Yunnan | Tibet |
|---|---|---|---|---|---|
| Sex | 1.340 | 1.313 | 1.358 | 1.595 | 1.184 |
| Age | 1.165 | 1.147 | 1.129 | 1.130 | 1.088 |
| BMI | 1.064 | 1.058 | 1.052 | 1.048 | 1.027 |
| Marital status | 1.018 | 1.015 | 1.037 | 1.041 | 1.011 |
| Educational attainment | 1.191 | 1.109 | 1.124 | 1.078 | 1.035 |
| Ethnicity | 1.298 | 1.001 | 1.001 | 1.077 | / |
| Annual income | 1.147 | 1.079 | 1.148 | 1.059 | 1.042 |

| Variable | Sichuan | Chongqing | Guizhou | Yunnan | Tibet |
|---|---|---|---|---|---|
| Smoking | 1.236 | 1.203 | 1.267 | 1.478 | 1.144 |
| Alcohol drinking | 1.171 | 1.089 | 1.091 | 1.191 | 1.052 |
| Sleep disorder | 1.012 | 1.038 | 1.006 | 1.017 | 1.022 |
| Physical activity | 1.036 | 1.044 | 1.029 | 1.044 | 1.007 |
| SBP | 1.673 | 1.599 | 1.801 | 1.721 | 1.888 |
| DBP | 1.584 | 1.498 | 1.731 | 1.637 | 1.833 |
| FPG | 1.067 | 1.063 | 1.039 | 1.037 | 1.033 |
| TG | 1.152 | 1.306 | 1.179 | 1.317 | 1.152 |
| TC | 1.171 | 1.151 | 1.228 | 1.216 | 1.233 |
| HDL_C | 1.222 | 1.330 | 1.174 | 1.304 | 1.195 |
| Calcium | 1.105 | 1.069 | 1.052 | 1.043 | 1.081 |
| Vitamin D | 1.077 | 1.052 | 1.040 | 1.025 | 1.076 |
| Dairy products | 1.032 | 1.045 | 1.043 | 1.022 | 1.011 |

Note: Values in the table are presented as adjusted Generalized Variance Inflation Factors ($GVIF^{1/(2\times df)}$), where df denotes degrees of freedom. Ethnicity was not included in the Tibet-specific model because all participants in the Tibet cohort were Tibetan and the variable therefore had no variation. All values are below 2.0.

**Table S4.** Summary of Brant-test diagnostics and PPOM specification in sensitivity analyses

| Analysis | Province | Global Brant *P* | Violating term(s) | Relaxed variable(s) |
|---|---|---|---|---|
| **Sensitivity analysis: intermediate-sample-size** | **Chongqing** | <0.001 | BMI (obesity); annual family income; education (medium); alcohol drinking; DBP | BMI; annual family income; **educational attainment**; alcohol drinking; DBP |
| | Guizhou | <0.001 | Age (>65 years); sex; BMI (overweight, obesity); SBP | Age; sex; BMI; SBP |
| | Yunnan | <0.001 | BMI (obesity); ethnicity; alcohol drinking; physical activity (medium, high) | BMI; ethnicity; alcohol drinking; physical activity |
| **Sensitivity analysis: women** | Sichuan | <0.0001 | Menopausal status (postmenopausal); BMI (overweight, obesity); ethnicity; vitamin D supplementation | Menopausal status; BMI; ethnicity; vitamin D supplementation |

| Analysis | Province | Global Brant *P* | Violating term(s) | Relaxed variable(s) |
|---|---|---|---|---|
| | **Chongqing** | <0.0001 | Age (45-65, >65 years); education (medium); alcohol drinking | Age; **educational attainment**; alcohol drinking |
| | Guizhou | 0.0398 | Menopausal status (postmenopausal); BMI (obesity); HDL-C | Menopausal status; BMI; HDL-C |
| | Yunnan | 0.0007 | BMI (overweight); ethnicity; alcohol drinking; physical activity (medium, high) | BMI; ethnicity; alcohol drinking; physical activity |
| | **Tibet** | 0.0002 | Age (45-65 years); menopausal status (postmenopausal); BMI (obesity); annual family income; education (high); FPG | Age; menopausal status; BMI; annual family income; **educational attainment**; FPG |
| **Sensitivity analysis: men** | Sichuan | <0.0001 | Age (>65 years); BMI (obesity); ethnicity; sleep disorder; FPG | Age; BMI; ethnicity; sleep disorder; FPG |
| | Chongqing | <0.0001 | BMI (obesity); annual family income | BMI; annual family income |
| | Guizhou | <0.0001 | BMI (overweight, obesity); SBP; DBP | BMI; SBP; DBP |
| | Yunnan | <0.0001 | Ethnicity; alcohol drinking; physical activity (high); calcium supplementation | Ethnicity; alcohol drinking; physical activity; calcium supplementation |
| | Tibet* | 0.0649 | BMI (obesity); DBP showed term-specific departures | None |
| **Sensitivity analysis: multiple imputation** | Sichuan | <0.0001 | BMI (overweight, obesity); ethnicity; sleep disorder; calcium supplementation; vitamin D supplementation | BMI; ethnicity; sleep disorder; calcium supplementation; vitamin D supplementation |
| | **Chongqing** | <0.0001 | Sex; BMI (obesity); education (medium); vitamin D supplementation | Sex; BMI; **educational attainment**; vitamin D supplementation |
| | Guizhou | <0.0001 | Age (>65 years); sex; BMI (overweight, obesity) | Age; sex; BMI |
| | Yunnan | <0.0001 | BMI (obesity); ethnicity; smoking; alcohol drinking; physical activity (medium, high) | BMI; ethnicity; smoking; alcohol drinking; physical activity |
| | **Tibet** | 0.0003 | Sex; BMI (obesity); annual family income; education (high) | Sex; BMI; annual family income; **educational attainment** |

Notes: The proportional-odds (PO) assumption was first assessed using the global Brant test. When the global Brant test yielded $P < 0.05$, a partial proportional-odds model (PPOM) was fitted; otherwise, the conventional PO model was retained. Term-specific Brant tests were then used to identify individual predictor terms violating the PO assumption, with "Violating term(s)" denoting terms with $P < 0.05$. Variables containing violating terms were allowed to have threshold-specific coefficients, and are listed under "Relaxed variable(s)." For multicategorical variables, violation by any indicator resulted in relaxation of the proportionality constraint for the variable as a whole. [*]For Tibetan men, the global Brant test was not significant ($P = 0.0649$); therefore, the conventional PO model was retained. PO, proportional odds; PPOM, partial proportional-odds model.

**Table S5.** Educational attainment and bone status in Chongqing: PPOM sensitivity analysis

| Variable | Coefficient | SE | *P* | OR (95% CI) | Term-specific Brant test *P* |
|---|---|---|---|---|---|
| Age(45,65] | 0.1796 | 0.0417 | <0.001 | 1.197 (1.103-1.299) | 0.5196 |
| Age(65,100] | 0.7549 | 0.0648 | <0.001 | 2.127 (1.874-2.415) | 0.2731 |
| Sex (Male) | 0.7796 | 0.0437 | <0.001 | 2.181 (2.002-2.375) | 0.2451 |
| BMI(Overweight):1 | -0.1566 | 0.0399 | <0.001 | 0.855 (0.791-0.925) | 0.7565 |
| BMI(Overweight):2 | -0.1463 | 0.0715 | 0.041 | 0.864 (0.751-0.994) | |
| BMI(Obesity):1 | -0.0982 | 0.0564 | 0.082 | 0.906 (0.812-1.012) | **0.0028** |
| BMI(Obesity):2 | 0.1595 | 0.0919 | 0.082 | 1.173 (0.98-1.404) | |
| Marital status(with a spouse) | -0.1978 | 0.0514 | <0.001 | 0.821 (0.742-0.908) | 0.5401 |
| Ethnicity (ethnic minority) | 1.4565 | 1.9290 | 0.450 | 4.291 (0.098-188.152) | 0.9317 |
| Annual income (>60,000 CNY/year):1 | -0.1013 | 0.0378 | 0.007 | 0.904 (0.839-0.973) | **0.0241** |
| Annual income (>60,000 CNY/year):2 | -0.2923 | 0.0727 | <0.001 | 0.747 (0.647-0.861) | |
| Education (Medium):1 | -0.0664 | 0.0418 | 0.112 | 0.936 (0.862-1.016) | **0.0275** |
| Education (Medium):2 | -0.2879 | 0.0700 | <0.001 | 0.75 (0.654-0.86) | |
| Education (High):1 | -0.3031 | 0.0638 | <0.001 | 0.738 (0.652-0.837) | 0.8740 |
| Education (High):2 | -0.3735 | 0.1225 | 0.002 | 0.688 (0.541-0.875) | |
| Smoking (Yes) | 0.1539 | 0.0481 | 0.001 | 1.166 (1.062-1.282) | 0.7623 |
| Alcohol drinking (Yes):1 | -0.1396 | 0.0377 | <0.001 | 0.87 (0.808-0.936) | **0.0398** |

| Variable | Coefficient | SE | *P* | OR (95% CI) | Term-specific Brant test *P* |
|---|---|---|---|---|---|
| Alcohol drinking (Yes):2 | -0.2955 | 0.0658 | <0.001 | 0.744 (0.654-0.847) | |
| Sleep disorder (Yes) | 0.0876 | 0.0345 | 0.011 | 1.092 (1.02-1.168) | 0.8076 |
| Physical activity(Medium) | -0.0806 | 0.0416 | 0.053 | 0.923 (0.85-1.001) | 0.3048 |
| Physical activity(High) | -0.1052 | 0.0514 | 0.041 | 0.9 (0.814-0.996) | 0.8028 |
| SBP | -0.0015 | 0.0013 | 0.266 | 0.999 (0.996-1.001) | 0.3522 |
| DBP:1 | 0.0102 | 0.0022 | <0.001 | 1.01 (1.006-1.015) | **0.0310** |
| DBP:2 | 0.0170 | 0.0030 | <0.001 | 1.017 (1.011-1.023) | |
| FPG | -0.0063 | 0.0114 | 0.578 | 0.994 (0.972-1.016) | 0.8566 |
| TG | 0.0038 | 0.0201 | 0.851 | 1.004 (0.965-1.044) | 0.1709 |
| TC | 0.0687 | 0.0194 | <0.001 | 1.071 (1.031-1.113) | 0.7097 |
| HDL_C | 0.1186 | 0.0511 | 0.020 | 1.126 (1.019-1.244) | 0.1820 |
| Calcium(Yes) | 0.1341 | 0.0590 | 0.023 | 1.143 (1.018-1.284) | 0.8277 |
| Vitamin D(Yes) | -0.2837 | 0.1306 | 0.030 | 0.753 (0.583-0.973) | 0.0738 |
| Dairy products | -0.0222 | 0.0073 | 0.002 | 0.978 (0.964-0.992) | 0.1479 |

Notes: The global Brant test was statistically significant ($P < 0.001$), indicating violation of the proportional-odds assumption; a fully adjusted partial proportional-odds model was therefore fitted. Reference categories: youngest age group, female sex, normal BMI, no spouse, Han ethnicity, annual family income <60,000 CNY/year, low educational attainment, no smoking, no alcohol consumption, no sleep disorder, low physical activity, no calcium or vitamin D supplementation. “:1” and “:2” correspond to the two cumulative logits in the PPOM model (normal vs. reduced or markedly reduced; normal or reduced vs. markedly reduced), respectively. ORs for continuous variables represent the change in odds associated with a 1-unit increase. ORs for continuous variables represent the change in odds associated with a 1-unit increase. ORs >1 indicate higher odds of being classified into a more reduced QUS-defined bone-status category, whereas ORs <1 indicate lower odds of being classified into a more reduced QUS-defined bone-status category. SE, standard error; OR, odds ratio; CI, confidence interval; BMI, body mass index; SBP, systolic blood pressure; DBP, diastolic blood pressure; FPG, fasting plasma glucose; TG, triglycerides; TC, total cholesterol; HDL-C, high-density lipoprotein cholesterol.

**Table S6.** Educational attainment and bone status in Guizhou: PPOM sensitivity analysis

| Variable | Coefficient | SE | *P* | OR (95% CI) | Term-specific Brant test *P* |
|---|---|---|---|---|---|
| Age (45,65]:1 | 0.5037 | 0.0464 | <0.001 | 1.655 (1.511-1.812) | 0.6551 |
| Age (45,65]:2 | 0.5360 | 0.1466 | <0.001 | 1.709 (1.282-2.278) | |
| Age (65,100]:1 | 1.4788 | 0.0706 | <0.001 | 4.388 (3.821-5.039) | **0.0109** |
| Age (65,100]:2 | 1.2990 | 0.1667 | <0.001 | 3.666 (2.644-5.082) | |
| Sex (Male):1 | 1.1102 | 0.0496 | <0.001 | 3.035 (2.754-3.345) | **<0.0001** |
| Sex (Male):2 | 1.9362 | 0.1182 | <0.001 | 6.932 (5.499-8.739) | |
| BMI (Overweight):1 | -0.1627 | 0.0419 | <0.001 | 0.85 (0.783-0.923) | **0.0011** |
| BMI (Overweight):2 | 0.2473 | 0.1171 | 0.035 | 1.281 (1.018-1.611) | |
| BMI (Obesity):1 | 0.1025 | 0.0578 | 0.076 | 1.108 (0.989-1.241) | **<0.0001** |
| BMI (Obesity):2 | 1.1560 | 0.1306 | <0.001 | 3.177 (2.46-4.104) | |
| Marital status (with a spouse) | -0.3385 | 0.0532 | <0.001 | 0.713 (0.642-0.791) | 0.5759 |
| Ethnicity (ethnic minority) | -1.3792 | 1.8968 | 0.467 | 0.252 (0.006-10.365) | 0.9306 |
| Annual income (>60,000 CNY/year) | 0.1605 | 0.0500 | 0.001 | 1.174 (1.065-1.295) | 0.9274 |
| Education (Medium) | 0.1407 | 0.0438 | 0.001 | 1.151 (1.056-1.254) | 0.5265 |
| Education (High) | 0.1601 | 0.0723 | 0.027 | 1.174 (1.018-1.352) | 0.5365 |
| Smoking (Yes) | 0.1420 | 0.0566 | 0.012 | 1.153 (1.032-1.288) | 0.6722 |
| Alcohol drinking (Yes) | -0.2094 | 0.0391 | <0.001 | 0.811 (0.751-0.876) | 0.4557 |
| Sleep disorder (Yes) | 0.0766 | 0.0355 | 0.031 | 1.08 (1.007-1.157) | 0.6585 |
| Physical activity (Medium) | -0.1673 | 0.0452 | <0.001 | 0.846 (0.774-0.924) | 0.8634 |
| Physical activity (High) | -0.3279 | 0.0512 | <0.001 | 0.72 (0.652-0.797) | 0.9722 |
| SBP:1 | 0.0031 | 0.0016 | 0.050 | 1.003 (1-1.006) | **0.0096** |
| SBP:2 | 0.0075 | 0.0028 | 0.007 | 1.008 (1.002-1.013) | |
| DBP | -0.0057 | 0.0026 | 0.029 | 0.994 (0.989-0.999) | 0.0784 |
| FPG | -0.0323 | 0.0133 | 0.015 | 0.968 (0.943-0.994) | 0.5591 |
| TG | -0.0164 | 0.0113 | 0.148 | 0.984 (0.962-1.006) | 0.3765 |
| TC | 0.1379 | 0.0217 | <0.001 | 1.148 (1.1-1.198) | 0.4370 |

| Variable | Coefficient | SE | *P* | OR (95% CI) | Term-specific Brant test *P* |
|---|---|---|---|---|---|
| HDL_C | -0.0338 | 0.0582 | 0.561 | 0.967 (0.863-1.083) | 0.0751 |
| Calcium (Yes) | 0.0534 | 0.0570 | 0.349 | 1.055 (0.943-1.18) | 0.8615 |
| Vitamin D (Yes) | -0.1070 | 0.1564 | 0.494 | 0.899 (0.661-1.221) | 0.2486 |
| Dairy products | -0.0139 | 0.0136 | 0.307 | 0.986 (0.96-1.013) | 0.6251 |

Notes: The global Brant test was statistically significant (*P* < 0.001), indicating violation of the proportional-odds assumption; a fully adjusted partial proportional-odds model was therefore fitted. Reference categories: youngest age group, female sex, normal BMI, no spouse, Han ethnicity, annual family income <60,000 CNY/year, low educational attainment, no smoking, no alcohol consumption, no sleep disorder, low physical activity, no calcium or vitamin D supplementation. “:1” and “:2” correspond to the two cumulative logits in the PPOM model (normal vs. reduced or markedly reduced; normal or reduced vs. markedly reduced), respectively. ORs for continuous variables represent the change in odds associated with a 1-unit increase. ORs >1 indicate higher odds of being classified into a more reduced QUS-defined bone-status category, whereas ORs <1 indicate lower odds of being classified into a more reduced QUS-defined bone-status category. SE, standard error; OR, odds ratio; CI, confidence interval; BMI, body mass index; SBP, systolic blood pressure; DBP, diastolic blood pressure; FPG, fasting plasma glucose; TG, triglycerides; TC, total cholesterol; HDL-C, high-density lipoprotein cholesterol.

**Table S7.** Educational attainment and bone status in Yunnan: PPOM sensitivity analysis

| Variable | Coefficient | SE | *P* | OR (95% CI) | Term-specific Brant test *P* |
|---|---|---|---|---|---|
| Age (45,65] | 0.5806 | 0.0455 | <0.001 | 1.787 (1.635-1.954) | 0.6821 |
| Age (65,100] | 1.7149 | 0.0664 | <0.001 | 5.556 (4.879-6.328) | 0.2858 |
| Sex (Male) | 1.2471 | 0.0524 | <0.001 | 3.48 (3.14-3.857) | 0.0625 |
| BMI (Overweight):1 | -0.2489 | 0.0400 | <0.001 | 0.78 (0.721-0.843) | 0.1783 |
| BMI (Overweight):2 | -0.1861 | 0.1073 | 0.083 | 0.83 (0.673-1.024) | |
| BMI (Obesity):1 | -0.3882 | 0.0708 | <0.001 | 0.678 (0.59-0.779) | **0.0120** |
| BMI (Obesity):2 | -0.0520 | 0.1876 | 0.782 | 0.949 (0.657-1.371) | |
| Marital status (with a spouse) | -0.3285 | 0.0535 | <0.001 | 0.72 (0.648-0.8) | 0.8272 |
| Ethnicity (ethnic minority):1 | -0.0598 | 0.0354 | 0.092 | 0.942 (0.879-1.01) | **<0.0001** |

| Variable | Coefficient | SE | *P* | OR (95% CI) | Term-specific Brant test *P* |
|---|---|---|---|---|---|
| Ethnicity (ethnic minority):2 | 0.4269 | 0.0941 | <0.001 | 1.533 (1.274-1.843) | |
| Annual income (>60,000 CNY/year) | 0.0677 | 0.0475 | 0.154 | 1.07 (0.975-1.174) | 0.0651 |
| Education (Medium) | -0.0483 | 0.0393 | 0.218 | 0.953 (0.882-1.029) | 0.7303 |
| Education (High) | -0.1363 | 0.1154 | 0.238 | 0.873 (0.696-1.094) | 0.8021 |
| Smoking (Yes) | 0.3144 | 0.0538 | <0.001 | 1.369 (1.232-1.522) | 0.0760 |
| Alcohol drinking (Yes):1 | -0.1708 | 0.0442 | <0.001 | 0.843 (0.773-0.919) | **0.0002** |
| Alcohol drinking (Yes):2 | -0.3198 | 0.0997 | 0.001 | 0.726 (0.597-0.883) | |
| Sleep disorder (Yes) | 0.0651 | 0.0340 | 0.055 | 1.067 (0.998-1.141) | 0.9879 |
| Physical activity (Medium):1 | -0.3113 | 0.0454 | <0.001 | 0.732 (0.67-0.801) | **0.0195** |
| Physical activity (Medium):2 | -0.5442 | 0.0985 | <0.001 | 0.58 (0.478-0.704) | |
| Physical activity (High):1 | -0.5924 | 0.0489 | <0.001 | 0.553 (0.502-0.609) | **0.0007** |
| Physical activity (High):2 | -0.9972 | 0.1265 | <0.001 | 0.369 (0.288-0.473) | |
| SBP | 0.0038 | 0.0014 | 0.006 | 1.004 (1.001-1.006) | 0.9931 |
| DBP | -0.0051 | 0.0022 | 0.022 | 0.995 (0.991-0.999) | 0.8069 |
| FPG | -0.0100 | 0.0134 | 0.455 | 0.99 (0.964-1.016) | 0.4747 |
| TG | 0.0094 | 0.0121 | 0.439 | 1.009 (0.986-1.034) | 0.6038 |
| TC | 0.0392 | 0.0188 | 0.037 | 1.04 (1.002-1.079) | 0.0633 |
| HDL_C | 0.3012 | 0.0480 | <0.001 | 1.351 (1.23-1.485) | 0.0535 |
| Calcium (Yes) | 0.2231 | 0.0450 | <0.001 | 1.25 (1.144-1.365) | 0.1550 |
| Vitamin D (Yes) | 0.0997 | 0.1406 | 0.478 | 1.105 (0.839-1.455) | 0.2504 |
| Dairy products | 0.0088 | 0.0097 | 0.367 | 1.009 (0.99-1.028) | 0.1484 |

Notes: The global Brant test was statistically significant ($P < 0.001$), indicating violation of the proportional-odds assumption; a fully adjusted partial proportional-odds model was therefore fitted. Reference categories: youngest age group, female sex, normal BMI, no spouse, Han ethnicity, annual family income <60,000 CNY/year, low educational attainment, no smoking, no alcohol consumption, no sleep disorder, low physical activity, no calcium or vitamin D supplementation. ":1" and ":2" correspond to the two cumulative logits in the PPOM model (normal vs. reduced or markedly reduced; normal or reduced vs. markedly reduced), respectively. ORs for continuous variables represent the change in odds associated with a 1-unit increase. ORs for continuous variables represent the change in

odds associated with a 1-unit increase. ORs >1 indicate higher odds of being classified into a more reduced QUS-defined bone-status category, whereas ORs <1 indicate lower odds of being classified into a more reduced QUS-defined bone-status category. SE, standard error; OR, odds ratio; CI, confidence interval; BMI, body mass index; SBP, systolic blood pressure; DBP, diastolic blood pressure; FPG, fasting plasma glucose; TG, triglycerides; TC, total cholesterol; HDL-C, high-density lipoprotein cholesterol.

**Table S8.** Province-specific educational attainment-bone status associations among women: PPOM sensitivity analysis

| Province | Variable | Coefficient | SE | *P* | OR (95% CI) | Term-specific Brant test *P* |
|---|---|---|---|---|---|---|
| Sichuan | Education (Medium) | 0.1580 | 0.0579 | 0.006 | 1.171 (1.046-1.312) | 0.8299 |
| | Education (High) | 0.3474 | 0.0917 | <0.001 | 1.415 (1.183-1.694) | 0.8660 |
| Chongqing | Education (Medium):1 | -0.0869 | 0.0579 | 0.133 | 0.917 (0.818-1.027) | **0.0223** |
| | Education (Medium):2 | -0.3878 | 0.1190 | 0.001 | 0.679 (0.537-0.857) | |
| | Education (High):1 | -0.1587 | 0.0936 | 0.090 | 0.853 (0.71-1.025) | 0.0792 |
| | Education (High):2 | 0.1201 | 0.2196 | 0.584 | 1.128 (0.733-1.734) | |
| Guizhou | Education (Medium) | 0.1705 | 0.0624 | 0.006 | 1.186 (1.049-1.34) | 0.7353 |
| | Education (High) | 0.0408 | 0.1117 | 0.715 | 1.042 (0.837-1.296) | 0.9833 |
| Yunnan | Education (Medium) | -0.0672 | 0.0638 | 0.292 | 0.935 (0.825-1.06) | 0.8621 |
| | Education (High) | -0.3267 | 0.2068 | 0.114 | 0.721 (0.481-1.082) | 0.9735 |
| Tibet | Education (Medium):1 | 0.2482 | 0.1717 | 0.148 | 1.282 (0.916-1.794) | 0.4246 |
| | Education (Medium):2 | 0.3697 | 0.3209 | 0.249 | 1.447 (0.772-2.715) | |

| Province | Variable | Coefficient | SE | *P* | OR (95% CI) | Term-specific Brant test *P* |
|---|---|---|---|---|---|---|
| | Education (High):1 | -0.2177 | 0.4489 | 0.628 | 0.804 (0.334-1.939) | **0.0079** |
| | Education (High):2 | 0.7813 | 0.7089 | 0.270 | 2.184 (0.544-8.765) | |

Notes: Low educational attainment was used as the reference category. Female-specific models included the same prespecified covariates as the primary analysis, except that sex was omitted because the analysis was restricted to women. Menopausal status, categorized as premenopausal, perimenopausal, or postmenopausal, was additionally included in all female-specific models. The global Brant tests rejected the proportional-odds assumption in all province-specific female models (all $P < 0.05$); therefore, partial proportional-odds models were fitted. ":1" and ":2" correspond to the two cumulative logits in the PPOM model (normal vs. reduced or markedly reduced; normal or reduced vs. markedly reduced), respectively. ORs for continuous variables represent the change in odds associated with a 1-unit increase. ORs >1 indicate higher odds of being classified into a more reduced QUS-defined bone-status category, whereas ORs <1 indicate lower odds of being classified into a more reduced QUS-defined bone-status category. SE, standard error; OR, odds ratio; CI, confidence interval; PPOM, partial proportional-odds model.

**Table S9.** Province-specific educational attainment-bone status associations among men: sensitivity analysis

| Province | Variable | Coefficient | SE | *P* | OR (95% CI) | Term-specific Brant test *P* |
|---|---|---|---|---|---|---|
| Sichuan | Education (Medium) | 0.1494 | 0.0553 | 0.007 | 1.161 (1.042-1.294) | 0.2101 |
| | Education (High) | 0.0403 | 0.0770 | 0.601 | 1.041 (0.895-1.211) | 0.1563 |
| Chongqing | Education (Medium) | -0.0453 | 0.0595 | 0.447 | 0.956 (0.851-1.074) | 0.1398 |
| | Education (High) | -0.3674 | 0.0883 | <0.001 | 0.693 (0.582-0.823) | 0.8674 |
| Guizhou | Education (Medium) | 0.1533 | 0.0646 | 0.018 | 1.166 (1.027-1.323) | 0.2852 |
| | Education (High) | 0.2088 | 0.1034 | 0.043 | 1.232 (1.006-1.509) | 0.9702 |

| Province | Variable | Coefficient | SE | *P* | OR (95% CI) | Term-specific Brant test *P* |
|---|---|---|---|---|---|---|
| Yunnan | Education (Medium) | 0.0809 | 0.0527 | 0.125 | 1.084 (0.978-1.202) | 0.4544 |
| | Education (High) | 0.1188 | 0.1518 | 0.434 | 1.126 (0.836-1.516) | 0.5376 |
| Tibet* | Education (Medium) | 0.2825 | 0.1597 | 0.077 | 1.326 (0.97-1.814) | 0.1062 |
| | Education (High) | 0.1551 | 0.3325 | 0.641 | 1.168 (0.609-2.241) | 0.7149 |

Notes: Low educational attainment was used as the reference category. Male-specific models included the same prespecified covariates as the primary analysis, except that sex was omitted because the analysis was restricted to men. The global Brant tests rejected the proportional-odds assumption in Sichuan, Chongqing, Guizhou, and Yunnan (all $P < 0.05$); therefore, partial proportional-odds models were fitted for these four provinces. *In Tibet, the global Brant test did not reject the proportional-odds assumption ($P = 0.0649$), and the conventional proportional-odds model was therefore retained. Because neither the medium- nor high-education indicator violated the proportional-odds assumption in any province, educational attainment was constrained to have a common coefficient across the two cumulative outcome thresholds, and a single OR is reported for each education category. ORs for continuous variables represent the change in odds associated with a 1-unit increase. ORs >1 indicate higher odds of being classified into a more reduced QUS-defined bone-status category, whereas ORs <1 indicate lower odds of being classified into a more reduced QUS-defined bone-status category. SE, standard error; OR, odds ratio; CI, confidence interval; PPOM, partial proportional-odds model.

**Table S10.** Selected sociodemographic characteristics of analytic participants and participants excluded for missing data

| Variable | Analytic Participants | Excluded Participants | SMD |
|---|---|---|---|
| **Age, years** | 51.54 ± 11.43 | 50.76 ± 11.79 | 0.067 |
| **Sex** | | | 0.108 |
| Female | 45681 (62.1) | 6263 (56.8) | |
| Male | 27919 (37.9) | 4770 (43.2) | |
| **Ethnicity** | | | 0.193 |
| Han Chinese | 41113 (55.9) | 7200 (65.3) | |
| Ethnic Minorities | 32487 (44.1) | 3833 (34.7) | |

| | | | |
|---|---|---|---|
| **Marital status** | | | 0.046 |
| Single | 7980 (10.8) | 1326 (12.0) | |
| Married | 65620 (89.2) | 9703 (87.9) | |
| Missing | 0 (0.0) | 4 (0.1) | |
| **Annual family income (CNY/year)** | | | 0.180 |
| <60000 | 53464 (72.6) | 7394 (67.0) | |
| ≥60000 | 20136 (27.4) | 3527 (32.0) | |
| Missing | 0 (0.0) | 112 (1.0) | |
| **Educational attainment** | | | 0.205 |
| Low | 39341 (53.5) | 4893 (44.3) | |
| Medium | 26674 (36.2) | 4444 (40.3) | |
| High | 7585 (10.3) | 1692 (15.3) | |
| Missing | 0 (0.0) | 4 (0.1) | |

Notes: Data are presented as mean ± standard deviation or n (%). Percentages were calculated using the total number of participants in each group. Standardized mean differences (SMDs) were used to assess differences between the analytic sample and participants excluded due to missing data. For categorical variables, the SMD represents the overall difference across all categories and is therefore reported only once for each variable. An absolute SMD < 0.10 was considered negligible, 0.10-0.20 indicated a small difference, and ≥ 0.20 indicated a potentially meaningful difference.

**Table S11.** Multicollinearity diagnostics for province-specific models after multiple imputation

| Variable | Sichuan | Chongqing | Guizhou | Yunnan | Tibet |
|---|---|---|---|---|---|
| Sex | 1.272 | 1.232 | 1.325 | 1.562 | 1.145 |
| Age | 1.101 | 1.089 | 1.071 | 1.083 | 1.028 |
| BMI | 1.020 | 1.010 | 1.006 | 1.007 | 1.007 |
| Marital status | 1.017 | 1.015 | 1.035 | 1.040 | 1.008 |
| Educational attainment | 1.189 | 1.107 | 1.123 | 1.076 | 1.035 |
| Ethnicity | 1.276 | 1.000 | 1.001 | 1.014 | 1.006 |
| Annual income | 1.148 | 1.080 | 1.148 | 1.058 | 1.042 |
| Smoking | 1.232 | 1.179 | 1.264 | 1.465 | 1.143 |

| Variable | Sichuan | Chongqing | Guizhou | Yunnan | Tibet |
|---|---|---|---|---|---|
| Alcohol drinking | 1.173 | 1.077 | 1.085 | 1.177 | 1.033 |
| Sleep disorder | 1.011 | 1.037 | 1.006 | 1.016 | 1.023 |
| Physical activity | 1.034 | 1.044 | 1.028 | 1.041 | 1.006 |
| Calcium | 1.088 | 1.044 | 1.041 | 1.030 | 1.083 |
| Vitamin D | 1.063 | 1.037 | 1.030 | 1.015 | 1.081 |
| Dairy products | 1.030 | 1.044 | 1.042 | 1.022 | 1.008 |

Note: Values in the table are presented as adjusted Generalized Variance Inflation Factors ($GVIF^{1/(2\times df)}$), where df denotes degrees of freedom. All values are below 2.0.

**Table S12.** Province-specific educational attainment-bone status associations after multiple imputation: PPOM sensitivity analysis

| Province | Variable | Coefficient | SE | *P* | OR (95% CI) | Term-specific Brant test *P* |
|---|---|---|---|---|---|---|
| Sichuan | Education (Medium) | 0.1002 | 0.0373 | 0.007 | 1.105 (1.028-1.189) | 0.3556 |
| | Education (High) | 0.0719 | 0.0540 | 0.183 | 1.075 (0.967-1.194) | 0.2005 |
| Chongqing | Education (Medium):1 | -0.0529 | 0.0356 | 0.138 | 0.949 (0.885-1.017) | **0.0182** |
| | Education (Medium):2 | -0.2481 | 0.0546 | <0.001 | 0.78 (0.701-0.868) | |
| | Education (High):1 | -0.2315 | 0.0515 | <0.001 | 0.793 (0.717-0.878) | 0.4856 |
| | Education (High):2 | -0.2864 | 0.0819 | <0.001 | 0.751 (0.64-0.882) | |
| Guizhou | Education (Medium) | 0.1432 | 0.0425 | <0.001 | 1.154 (1.062-1.254) | 0.4336 |
| | Education (High) | 0.1538 | 0.0703 | 0.029 | 1.166 (1.016-1.338) | 0.7289 |
| Yunnan | Education (Medium) | -0.0489 | 0.0374 | 0.191 | 0.952 (0.885-1.025) | 0.7650 |
| | Education (High) | -0.1611 | 0.1101 | 0.143 | 0.851 (0.686-1.056) | 0.5793 |

| Province | Variable | Coefficient | SE | *P* | OR (95% CI) | Term-specific Brant test *P* |
|---|---|---|---|---|---|---|
| Tibet | Education (Medium):1 | 0.2737 | 0.1030 | 0.008 | 1.315 (1.074-1.609) | 0.5004 |
| | Education (Medium):2 | 0.1478 | 0.1919 | 0.441 | 1.159 (0.796-1.688) | |
| | Education (High):1 | -0.0678 | 0.2374 | 0.775 | 0.934 (0.587-1.488) | **0.0080** |
| | Education (High):2 | 0.7255 | 0.3546 | 0.041 | 2.066 (1.031-4.139) | |

Notes: Low educational attainment was used as the reference category. The models were adjusted for the same prespecified covariates as the primary province-specific analyses. The global Brant tests rejected the proportional-odds assumption in all province-specific models (all $P < 0.05$); therefore, partial proportional-odds models were fitted. ":1" and ":2" correspond to the two cumulative logits in the PPOM model (normal vs. reduced or markedly reduced; normal or reduced vs. markedly reduced), respectively. ORs for continuous variables represent the change in odds associated with a 1-unit increase. ORs >1 indicate higher odds of being classified into a more reduced QUS-defined bone-status category, whereas ORs <1 indicate lower odds of being classified into a more reduced QUS-defined bone-status category. SE, standard error; OR, odds ratio; CI, confidence interval; PPOM, partial proportional-odds model. Estimates were derived from sensitivity analyses using the multiply imputed dataset generated by Multiple Imputation by Chained Equations (MICE).
